# Diseases of the Viscera

**Published:** 1876-06

**Authors:** 


Diseases of the Viscera as Related to Pain or Tenderness of Certain Portions of the Spinal Cord.—Leyden (Journal of Nervous and Mental Diseases, Jan., 1876) says that affections of the stomach most frequently have their dorsal point at the fourth dorsal vertebra, affections of the heart at the fourth and fifth dorsal vertebræ, while those most often tender in biliary colic are the eighth and ninth dorsal. These visceral affections are frequently combined with neuralgias; for example, many heart affections, particularly palpitations, and angina pectoris, being accompanied by intercostal and brachial neuralgias. Traube has shown, in respect to gastric diseases, that intercostal neuralgias are often a prominent symptom of ulcer of the stomach. An analogous significance has spinal pain. Sometimes it is extremely severe, and so far as my observation goes to show, it most frequently occurs in connection with those gastric affections which lead to the rapid development in and eructations of gas from the stomach. In an aged lady, who sometimes exhibited nervous symptoms, I saw, during an exacerbation of a chronic gastric catarrh, such severe pain and tenderness of the spinous process of the fourth and fifth dorsal vertebra, that the patient bent backwards in distress at the slightest touch. With the improvement of the stomach disorder there was a corresponding diminution of the symptoms of spinal irritation. In another patient, a healthy looking girl, who suffered with a periodically remitting and exacerbating gastralgia, with formation of gas in the stomach, the exacerbation was introduced each time by extreme sensibility of a space along the spine, extending from the fifth to the seventh dorsal vertebra, and with a feeling of fatigue in the back, and radiating pains in the neck and along the arms as far as the elbows.—*Detroit Review*, May, '76.

